# MicroRNAs: Mediators and Therapeutic Targets to Airway Hyper Reactivity After Respiratory Syncytial Virus Infection

**DOI:** 10.3389/fmicb.2018.02177

**Published:** 2018-09-11

**Authors:** Shuwen Feng, Dongxin Zeng, Junwen Zheng, Dongchi Zhao

**Affiliations:** Department of Pediatrics, Children’s Digital Health and Data Center, Zhongnan Hospital of Wuhan University, Wuhan, China

**Keywords:** microRNAs, airway hyper reactivity, immune dysfunction, respiratory syncytial virus, immune tolerance

## Abstract

Respiratory syncytial virus (RSV) is the most important pathogen correlated to the first-time infant wheezing and later recurrence after its primary infection. RSV infection promotes the bronchial smooth muscle sensitivity to leukotrienes (LTs) in acute stage, causes the extensive inflammatory reaction and the aggregation of Th2-like cells during respiratory tract obstruction. Infants and young children infected with RSV exhibit an increased susceptibility to the exposure of exogenous allergens, easy to suffer from the recurrent wheezing, which prompts that the body is still in a state of inflammation or immunological bias. However, the pathological mechanism is unclear. The recent researches demonstrate that abnormal expression of non-coding microRNAs (miRNAs) can be detected from the peripheral blood and airway tract epithelial of RSV infected infants, which participate the regulation of immune cells polarization and LTs synthesis. Improving the immune tolerance can significantly relieve the airway inflammation and broncho-spasm caused by RSV. In this review, we discuss recent advances in understanding the mechanism of RSV-induced inflammatory reaction and immune dysfunction leading to airway hyper-reactivity. Further, we summarize the potential molecular basis that, in this process, miRNAs, which are produced by airway epithelial cells or peripheral blood mononuclear cells, directly or in the form of exosome to regulate the inflammation programs as well as the function, differentiation and proliferation of immune cells. miRNAs may become a potential bio-marker of detecting severe RSV infection and a novel target of early intervention and therapeutic strategy in recurrent wheezing or asthma related to RSV infection.

## Introduction

Respiratory syncytial virus (RSV), a single-stranded, negative-sense RNA virus belonging to the genus *Orthopneumovirus* of the new family *Pneumoviridae*, is the most common etiological agent for acute lower respiratory infection (ALRI) in infants aged less than 24 months. The clinical course contains both the wheezing (bronchiolitis) during acute infection period and the secondary airway hyperactivity (AHR) in the late life (Bisgaard, 2003; Zheng et al., 2015a; Frias et al., 2018; Petrarca et al., 2018). The infection of RSV is restricted in airways, viral replication results in the recruitment of inflammatory cells, releasing inflammatory factors which contribute to the airway edema, sputum accumulation, smooth muscle spasm, leading to the airway obstruction manifested as wheezing (Johnson et al., 2007; Shirey et al., 2014). Furthermore, in infants infected with RSV, the T helper 17 cell (Th17)/regulatory T-cell (Treg) imbalance, and the over-expression of Th2-like cytokines make the airway inflammatory reaction getting worse, particularly in preterm newborns, RSV infections are more likely to become severe cases (Mangodt et al., 2015).

Usually, AHR lasts for months or even years after the acute infection stage is one of the significant clinical features in infants primary challenged by RSV, who exhibit an increased susceptibility to the exogenous allergens and reinfection to evoke asthma, especially in those severe enough to cause hospitalization, which is highly associated with the development of asthma and allergic sensitization up to age 7 (Sigurs et al., 2000; Nenna et al., 2015). However, the pathological mechanism could not be appropriately explained so far. In recent years, the use of leukotriene receptor antagonist (LTRA) to prevent asthmatic attack has got certain effect (Bisgaard, 2003), which suggests that an alteration of leukotriene receptors (TLRs) expression pattern, possible the up-regulation of LTR in smooth muscle cells and enhanced sensitivity to leukotrienes (LTs), may be an important event that could contribute to the development of the histopathology in RSV infected individuals (Liu et al., 2015). Wheezing induced by primary RSV infections in early childhood is specifically time-ordered, it is quite often before 2 years old, then reduced, and obviously less occurs after 5, which is in agreement with the process of lymphocytes differentiation and functional maturation during childhood (Simoes et al., 2018). MicroRNAs (miRNAs) are small, non-coding, regulatory RNAs which can induce message RNA (mRNA) degradation or translational repression of their target genes. miRNA species have recently emerged as gene expression regulators that play a modulatory role in virus infection by modifying host responses in inflammatory and immune cells as well as airway epithelial cells (Globinska et al., 2014; Werz et al., 2017). It has been shown that abnormal expression of miRNAs can be detected from the peripheral blood and airway tract of RSV infected infants, involved in the regulation of 5-lipoxygenase (5-LO) activity and lymphocyte differentiation (Othumpangat et al., 2012; Simpson et al., 2014; Busch et al., 2015; Su et al., 2016; Wang et al., 2017; Werz et al., 2017).

Given this, it could be inferred that, during an interval of time after acute infection, RSV could trigger inflammatory programs, and subsequently comes the decreased host immune tolerance and hyper-function of Th2-like cells, which might be the pathological basis of the chronic airway inflammation. In view of miRNA plays an important role in RSV-related AHR, further studies are required to identify the possible molecular basis of abnormal behaviors of immune and inflammatory cells, in which miRNAs could act as inflammatory mediators and can be used as the potential bio-markers as well as the therapeutic targets.

## RSV-Induced Inflammatory Reaction and Immune Dysfunction

Globally, RSV is the most common pathogen identified in infants with ALRI (Shi et al., 2015). The feature in histopathological studies of children with RSV-ALRI is mucus inflammatory exudation and small airway filled with dense plugs composed of mucus, fibrin, and debris from leukocytes and sloughed bronchial epithelial cells, which is the primary cause of airway stenosis and obstruction, smooth muscle spasm of bronchioles also plays a role (Cortjens et al., 2016). Activation and release of inflammatory mediators promoted by virus infection could induce enhanced immune reaction, which brings about immune dysfunction and further exacerbated immunopathology (Schmidt et al., 2018).

During the activation of innate immunity by airway epithelial cells (AECs) upon RSV infection, AECs can sense RSV components through pattern recognition receptors (PRRs), including Toll-like receptors (TLRs) and retinoic acid-inducible gene I (RIG-I)-like receptor (RLR) family members which are coupled to various pathways that control the activation of the corresponding transcription factors, such as IRF-3 and NF-kB (Zheng et al., 2015b; Tian et al., 2017). Subsequently, the generation of an early innate immune response, including secretion of cytokines and antiviral molecular, is induced (Rossi and Colin, 2015; Lay et al., 2016). A serious of studies have shown that RSV infection stimulates RIG-I and TLR3 in AECs to produce thymic stromal lymphopoitein (TSLP), which enhances the immune response mediated by Th2 cells and promotes allergic inflammation and asthma (Lee et al., 2012; Feng et al., 2017). TSLP, an interleukin-7 (IL-7)-like cytokine derived from epithelial cells, is considered as a master switch in Th2 mediated immune responses, and is believed to play a key role in allergic asthma. It could promote the cytokine production in mast cells and the maturation of dendritic cells (DCs) during the sensitization/priming stage of innate and adaptive allergic responses, while it supports Th2 CD4+T-cell proliferation and also induces cytokine production during the challenge stage (Ziegler and Liu, 2006). In the TSLP/DC/OX40L pathway, TSLP triggers the Th2 mediated allergic cascade by induction of OX40-ligand (OX40L) on DCs, may contribute to asthma pathogenesis and airway inflammation by modulating level of CD4+CD25+Treg cells and the profile of inflammatory cytokines (Murakami-Satsutani et al., 2014; Feng et al., 2017). In the process of phagocytic recruitment and lymphocytic aggregation, virus could induce host cells to produce non-coding RNAs which interfere with the post-transcriptional biological activity of host mRNA, and also play an important role in the form of exosome to regulate target genes via blood circulation (Anderson et al., 2016; Hasegawa et al., 2018). miRNAs exert an extensive regulatory influence on the production of inflammatory mediators, the stimulation of pattern recognition receptors expressions in epithelial cells, as well as the oriented differentiation of immune cells (Lay et al., 2016; Peng et al., 2018).

**FIGURE 1:**
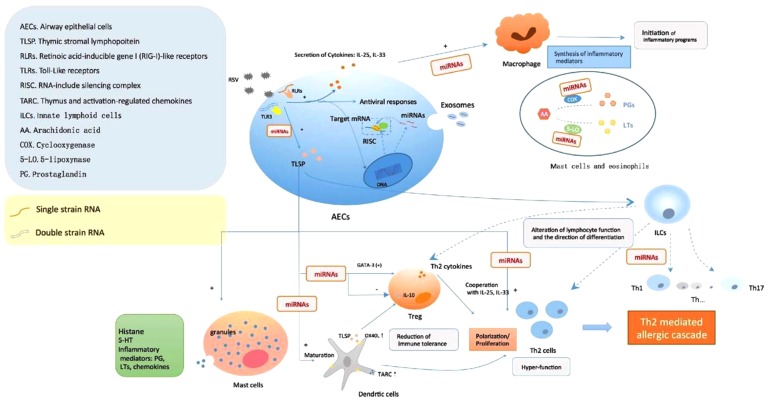
Respiratory syncytial virus (RSV) infection causes AHR formation mediated by miRNAs. The viral single-stranded RNAs and double-stranded RNAs components during RSV replication were recognized by pattern recognition receptors in AECs, subsequently induce the secretion of cytokines and antiviral responses. In the meanwhile, the upregulation/downregulation of miRNAs began to take part in regulating the expression of target genes, such as TSLP, one of the most important factors in allergic inflammation, which might have an impact on multiple inflammation and immunologic process: degranulation of mast cells, activation and maturation of DCs, alteration of lymphocyte function and differentiation, weakness of Treg cells, polarization/proliferation of Th2 cells, etc. Furthermore, miRNAs also played a role in the activation of monocyte-macrophage via interfering PGs and 5-LO synthesis to produce inflammatory mediators. All these led to the initiation of aberrant inflammatory programs and immune dysfunction correlated to AHR post-RSV infection.

The underlying mechanisms contributed to asthma in children who underwent RSV wheezing episodes in early life are proposed. Firstly, RSV infection could induce a Th2-like effector phenotype in Treg cells, attenuating their capacity to promote tolerance to inhaled allergens by means of GATA-3 activation and Th2 cytokine production in FOXP3+ Treg cells (Chauhan et al., 2015; Hirose et al., 2017; Schmidt et al., 2018). Simultaneously, RVS may also induce the secretion of TSLP in epithelial cells, in addition to the possible role in RSV immunopathology, epithelium-derived cytokines IL-25 and IL-33 are also associated to an allergic Th2 response (Lay et al., 2016). Furthermore, the enhanced expression of thymus and activation-regulated chemokines (TARC) and OX40L of mature DCs activated by TSLP could promote Th2 polarization/proliferation in thymus and exacerbate the local inflammatory response (Hirose et al., 2017). In the meanwhile, TSLP directly or selectively impairs IL-10 production of FOXP3+ Treg cells and inhibited their suppressive activity (Chauhan et al., 2015). Therefore, initiation of inflammation program, hyper-function of Th2-like cells, as well as decreased immune tolerance, might be the crucial links to wheezing recurrence correlated to RSV infection, while miRNAs participate the regulation of both T-cells function and 5-LO synthesis procedures (**Figure 1**).

## RSV Infection Regulates Inflammation Mediated by miRNAs

Multiple non-coding RNAs take part in the regulation of post-transcriptional activity, of which, miRNAs are small, single-stranded, regulatory RNAs. With a length of 19–24 nt, it mediates the post-transcriptional silence of its target genes through incorporation into the RNA-include silencing complex (RISC) and then directing this complex to the 3′untranslated region (3′UTR) or open reading frame (ORF) of the target gene’s mRNA (Ross et al., 2007). For most interactions, miRNAs act as rheostats to make optimum-scale adjustments to specific protein output (Baek et al., 2008).

### miRNAs-Related Regulation of LTs Bio-Activity

Respiratory syncytial virus infection causes cysteinyl leukotrienes (CysLTs) increase in the bronchoalveolar lavage fluids and lung tissue (Musiyenko et al., 2009). Subsequently, sensitized with mite allergen, enhanced the allergic inflammation following allergen challenge, and this was accompanied by elevations in lung dendritic cells and CysLTs (Werz et al., 2017). Leukotrienes are lipid mediators produced from arachidonic acid (AA) with a broad variety of bioactivities in allergic inflammation and immune responses, which are over 1000 times more powerful than histamine (Liu et al., 2015). LTs are divided into two types, one is LTB4, considered as a potent chemoattractant for most subsets of leukocytes, whereas the another CysLTs are potent bronchoconstrictors that have effects on airway remodeling (Matsuse et al., 2007; Liu and Yokomizo, 2015). LTs exert their biological effects by binding to LTRs, to induce smooth muscle spasm, increased microvascular permeability, mucosal edema, mucous hypersecretion, migration of granulocytes to lung and their adhesion to endothelium, degranulation, release of lysosomal enzymes, etc., which are of vital importance in asthma airway inflammation. LTRs belong to G protein-coupled receptors (GPCRs), produced predominantly by immune and inflammatory cells, their expressions have also been detected in airway smooth muscle cells, endothelial cells as well as peripheral tissues and organs (Wang et al., 2014). In response to various biological stimuli, arachidonic acid is released from membrane phospholipids and transformed into LTs via multistep enzymatic reactions (Liu and Yokomizo, 2015). Arachidonic acid is first oxidized at the C-5 position by the dual enzymatic activity of 5-LO to yield 5-hydroxyperoxyeicosatetraenoic acid (5- HpETE) followed by an unstable intermediate, leukotriene A4 (LTA4). LTA4 is either converted to LTB4 by or conjugated to reduced glutathione to produce CysLT (LTC4). LTC4 is then exported from the cell and converted to LTD4 and LTE4, the most stable CysLTs, by extracellular peptidases. Among the multiple process of enzymatic reactions, 5-LO is the key enzyme which has been most widely studied, expressed mainly in granulocytes, macrophages and mast cells, catalyzing the conversion from arachidonic acid to LTs (Busch et al., 2015).

**FIGURE 2:**
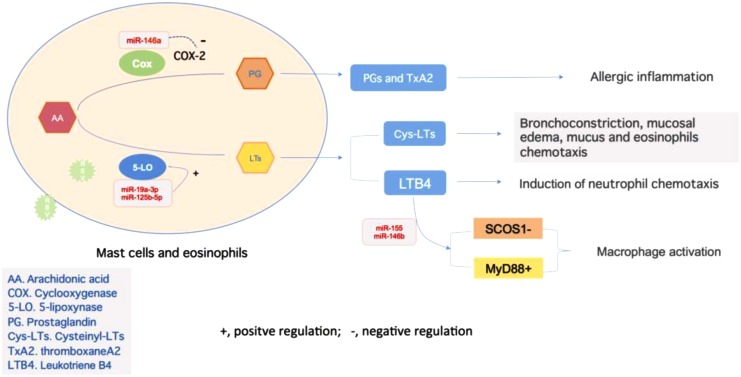
Regulations of inflammation reaction mediated by miRNAs. miR-146a negatively regulates the Cox-2 activity to influence the alteration from AA to PG, and later become TxA2, which is related to allergic inflammation. On the other hand, activity of 5-LO is under the regulation of miR-19a-3p and miR-125b-5p, which promote the production of LTs. These two types of LTs, CysLTs, and LTB4, act as bronchoconstrictors or chemoattractant to take an essential part in inflammation. The latter one could also promote the expression of miR-155 in macrophage, which target on both SCOS1 and MyD88 to control macrophage activation and airway remodeling.

Regulation of LTs bio-activity mainly includes two kinds of pathway: LTRAs and 5-LO inhibitors (Werz et al., 2017). The former blocks the organ response to LTs via selective binding with receptors in bronchial smooth muscles and competitively inhibiting effects of CysLTs, such as montelukast, showed the effect on controlling wheeze induced by RSV infection (Peng et al., 2014). The latter inhibits LTs synthesis via interfering 5-LO pathway (Liu et al., 2015). miRNAs modulate LTs synthesis by regulating 5-LO activity, and the effect of miRNAs derived from different cell lines seem to be cell type- and stimulus-specific, which might contribute to maintain physical function of LTs in normal circumstances. Busch et al. (2015) had revealed that the miRNA-19a-3p and miR-125-5p are involved in the regulation of leukocyte functions and immune responses by regulation 5-LO transcriptional efficiency (**Figure 2**). Antagonizing miR-19a-3p in PHA-stimulated T cells leads to an increase in 5-LO mRNA expression, and treatment of differentiated monocytic MM6 cells with antagomirs for miR-19a-3p and miR-125b-5p also increases 5-LO protein expression. And upregulation of LTRs was found in both human allergic nasal mucosa and asthmatic bronchial mucosa (Zhu et al., 2005; Shirasaki et al., 2013). LTB4, via leukotriene receptor 1 signaling, increases macrophage expression of miR-155, miR-146b, and miR-125b, in which, LTB4-mediated miR-155 generation was attributable to the protein-1 activation. Antagomirs against miR-155 and miR-146b prevented both the LTB4-mediated decrease in SOCS-1 and increase in MyD88, in which controls macrophage activation (Wang et al., 2014).

These researches above imply that RSV infection could interfere miRNAs expression to regulate LTs synthesis, which mediates airway hyperresponsiveness (Zago et al., 2014). In primary RSV infection, RSV could induce the expression of specific miRNAs which lead to upregulation of LTs bio-activity, contributing to inflammatory reactions and wheezing during acute infectious period. Continuous impacts may exist after primary infection, probably the upregulation of LTRs or influence on differentiation and function of immunocytes, which brings about chronic airway inflammation. Further studies are required to explore mechanisms of RSV-induced miRNAs expression and AHR.

### miRNAs Participate in Regulation of Innate Immune

miRNAs could take part in the regulation of early innate immune and initiation of inflammatory responses. Of which, macrophages are the major cellular component of the innate immune system, and play an important role in the recognition of microbes, particulates, immunogens and the regulation of inflammatory responses (Pappas et al., 2013). The central conserved region of RSV G protein modulates virus replication and the pattern of host gene expression by downregulating miRNAs let-7i to active IFN-λ secretion, which could induce innate antiviral responses in epithelial cells (Bakre et al., 2017). It has been demonstrated that miRNAs regulate M1 and M2 macrophage polarization through targeting adaptor proteins and transcription factors in various pathways to participate in inflammatory program modulation (Banerjee et al., 2013; Wang et al., 2014; Essandoh et al., 2016; Bras et al., 2017; Self-Fordham et al., 2017). Besides, miR-27a could negatively regulate the IL-10-dependent signal transducer and activator of transcription 3 (STAT3) phosphorylation, thereby relieve TLR2 and TLR4 driven inflammatory responses (Xie et al., 2014). STAT3 drives the development of Th17 cells and the cytokine production by Th2 and Th17 cells, and the activation of STAT3 pathway is closely related to the development of airway inflammation, which contribute a lot to asthma (Gavino et al., 2016). miR-19b can reduce airway remodeling, airway inflammation, and degree of oxidative stress by inhibiting STAT3 signaling through TSLP downregulation in a mouse asthma model (Ye et al., 2017). In addition, studied show that miRNAs modulate DCs development as well as their maturation process, antigen presentation capacity and cytokine release to take part in immune regulation, which link the innate and adaptive immune systems (Smyth et al., 2015).

## Alteration of Immunocyte Function Mediated by miRNAs

**FIGURE 3:**
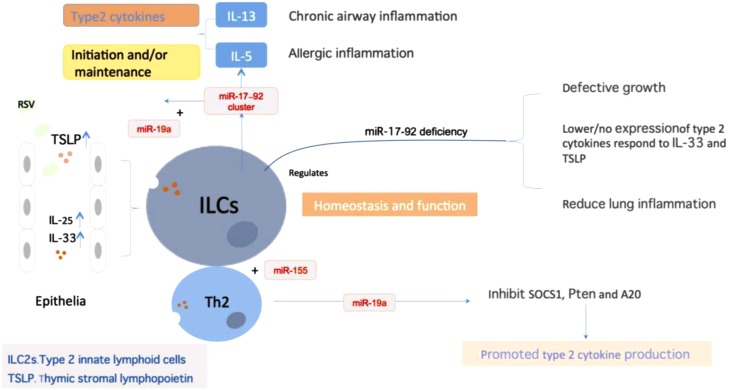
miRNAs regulate ILC2 homeostasis and function. miRNA expression was essential in maintaining ILCs homeostasis and function, particularly the miR-17∼92 cluster. Epithelium-derived cytokines IL-33, IL-25, and TSLP could activate ILCs to promote type2 cytokines production, which plays a role in the initiation and maintenance of chronic airway inflammation. During the process, miR-17∼92 promoted IL-13 and IL-5 production in response to IL-33 and ILC2-driven type 2 inflammation. miR-17∼92 deficiency will result in the defective growth in ILCs, decrease the respond to IL-33 and TSLP, and reduced lung inflammation. Besides, miR-155 is indispensible for the development of ILCs-homologous Th2 cells. miR-19a could promote type2 cytokine production of Th2 cells through targeting of the signal inhibitors Pten, SCOS1 and A20.

Respiratory syncytial virus replication could influence the expression of multiple genes related to immunity via miRNAs regulation, leading to alteration of lymphocytic function (Anderson et al., 2016). miRNAs are crucial regulators of type 2 innate lymphoid cells (ILC2s) biological function that mediate similar but non-identical programs of post-transcriptional gene regulation in innate and adaptive lymphocytes. It was identified in mice that the miR-17∼92 cluster is required to maintain ILC2 homeostasis and function *in vivo* (**Figure 3**). The over-expression of miR-17∼92 could promote the ILC2s production of IL-13 and IL-5 in response to IL-33 driven inflammation, and these cells could be detected in sputum and bronchoalveolar lavage (BAL) in human asthma (Singh et al., 2017). Activation of ILC2s in responds to IL-33, IL-25, and TSLP act as a role in the initiation and maintenance of chronic airway inflammation. Mice lacking of miR-17∼92 expression exhibit growth defect in ILC2s and decreased cytokine expression in respond to IL-33 and TSLP, which could relieve the allergic lung inflammation induced by allergens. Among them, miR-19a augments Th2 cytokines production and allergic inflammation via coordinate regulation of cytokine and antigen receptor signaling pathways [NF-κB, JAK-STAT, and PI(3)K] (Simpson et al., 2014). Another research suggested that tight and fine regulation of miR-23∼27∼24 clusters in T cells is required to maintain optimal effector function and to prevent aberrant immune responses. miR-24 and miR-27 collaboratively limit Th2 responses through targeting IL-4 and GATA3 in both direct and indirect manners. Although over-expression of the entire miR-23 cluster negatively impacts other Th lineages, enforced expression of miR-24, in contrast to miR-23 and miR-27, actually promotes the differentiation of Th1, Th17, and induced regulatory T cells (Cho et al., 2016). miR-31 increases the sensitivity of T cells to type I interferon, which interferes with effector T cell function and increases the expression of several proteins related to T cell dysfunction (Moffett et al., 2017). The nasal epithelial cell-derived miR-146a could induce the expression of IL-10 in monocytes, which suppresses the activities of CD4+ effector T cells and the Th2 polarization, and relieves allergic reactions induced by virus (Luo et al., 2015). Besides, upregulation of let-7d can be detected in epithelial cells of nasal mucosa in children with RSV infection (Inchley et al., 2015). In the meanwhile, let-7 miRNAs, possibly in the manner of exosome, play a significant role in the regulation of allergic airway inflammation by regulating the expression of IL-13 in Th2 cells (Kumar et al., 2011; Panganiban et al., 2012).

Our former research demonstrated that differential expression of miRNAs existed in the peripheral blood of infants with RSV infection, including the upregulation miRNAs (miR-106b-5p, miR-181a-5p, miR-20b-5p, miR-342-3p, and miR-652-3p) and the downregulation miRNAs (miR-122-5p, miR-320e, miR-320d, miR-877-5p, miR-92b-5p, and let-7c-5p) (Wang et al., 2017). Among them, miR-92b-5p and let-7c-5p have been reported in relation to G1/S and G2/M in cell circle, and they might regulate cell proliferation by targeting cyclins such as E2F1, CcnE, Cdc6, CcnB1, and Cdc25C (Markopoulos et al., 2017). RSV replication induces let-7i upregulation in epithelial cells while let-7b upregulation in DCs. And it appears to manipulate host pathways and nuclear transcription related to proliferation and differentiation through regulation of expression of these miRNAs in relation to the interferon response (Thornburg et al., 2012). Abnormal expression of miR-let-7 family in epithelial cells and lymphocytes might be closely related to airway inflammation and lymphocyte dysfunction during RSV infection. From the above, the physical expression of miRNAs is of vital importance in maintaining immunocyte function in lung and regulating Th2 cell homing. Aberrant expression of miRNAs evoked by viral infection, involving gene expression related to regulating immunocyte differentiation and immune tolerance, is in great association with asthma induction. miRNAs associated to airway hyperresponsiveness propelled by RSV infection are shown in **Table 1**.

**Table 1:** miRNAs distribution and function related to ARH caused by RSV infection.

miRNA classification	Cell type	Target	Function	Reference
miR-let-7f and miR-24	Epithelial cells, Calu-3 cell	IFNλ+	Virus replication and host gene expression pattern	Simpson et al., 2014
miR-221	Bronchial epithelial cells	NGF and TrKA	Modulates RSV replication	Othumpangat et al., 2012
miR-124, miR-24, and miR-744	A549	P38,MAPK-	Inhibits of Respiratory Virus Infection	McCaskill et al., 2017
miR-19a-3p and miR-125b-5p	Hematopoietic cell	5-LO	5-LO expression	Busch et al., 2015
miR-674-5p	Hepatocyte	5-LO	Harbors a potential binding region for 5-LO	Su et al., 2016
miR-155, miR-146b, and miR-125b	Macrophage	SOCS1-, MyD88+	Macrophage activation	Wang et al., 2014
miR-195	Macrophage	–	Inhibits TLR2 pathway	Bras et al., 2017
miR-125a-5p	Macrophage	KLF13	M2 polarization	Banerjee et al., 2013
miR-27a	Macrophage	IL-10, STAT3	Inhibits STAT3 phosphorylation	Xie et al., 2014
miR-19b	–	TSLP, STAT3	Inhibits STAT3 through TSLP	Ye et al., 2017
miR-146a	Monocyte	IL-10	Induces IL-10	Luo et al., 2015
	Fibroblasts	NF-kappaB	Suppress COX-2 protein expression	Zago et al., 2014
miR17∼92	ILCs	–	Proliferation, differentiation of T and B cells	Simpson et al., 2014; Singh et al., 2017
miR-19a, miR-19b	ILCs, Th2	A20, SOCS1, Pten	Promotes IL-13 and IL-5 production	Singh et al., 2017
miR-23∼27∼24	T lineages		Regulates Th 2 immunity	Cho et al., 2016
miR-24, miR-27	Th2	IL-4, STAT3	Limits Th2 response	Xie et al., 2014
miR-23 and miR-27	T lineages	TGF-β	Limits the differentiation of Th17 and iTreg	Cho et al., 2016
miR-24	T lineages	Smad7, TGF-β	Promotes the differentiation of Th17 and iTreg	
miR-31	CD8+ T cells	NFAT	Increases the sensitivity of T cells to type I IFN	Moffett et al., 2017
miR-142-3p	CD25+ CD4 T Cells	Glycoprotein A	Reduces immune tolerance	Zhou et al., 2013
miR-let-7 family	Epithelial cells, DCs, etc.	–	Regulates allergic inflammation	Roush and Slack, 2008; Kumar et al., 2011; Lee et al., 2016;


## miRNAs Are Involved in Lymphocyte Proliferation and Differentiation

Viral infection also influences host lymphocytes polarization mediated by miRNAs (Ren et al., 2016; Haralambieva et al., 2018). Studies on involvement of miRNAs in lymphocyte differentiation, maturation, proliferation and apoptosis have made some progress, but the mechanism is still not very clear (Kroesen et al., 2015). Elevating evidences suggest that an expanding list of individual miRNAs and co-expressed miRNA clusters have been shown to have significant effects on T cell fate decisions and immune functions: miR-21, miR-155, and miR-17∼92 participate in T cell differentiation, activation and lifespan, and miR-23, ∼24, ∼27 family is involved in effector T cell differentiation and function maturation (Baumjohann and Ansel, 2013; Jeker and Bluestone, 2013; Cho et al., 2016). miR-142-3p regulates CD4+CD25+T cell proliferation by targeting the expression of glycoprotein A repetitions predominant, then inhibits Treg cell proliferation and reduces immune tolerance (Zhou et al., 2013). The adaptive immune response mediated by central immune organ is also under the regulation of miRNA. For instance, miR-155 is engaged in antigen presentation and B and T cell development. Differential expression of miRNAs exists in different stage of differentiation of the same cell. miR-150 has been only found in mature resting T and B lymphocytes, it is less or not expressed in their precursors. Memory T lymphocytes showed high expression of miR-146a, while less expressed in prime T cells, which suggests miRNAs participating in maintaining specific cellular immune. Moreover, experimental evidence suggests that miR-146a targets the Fas-associated death domain and affects activation-induced cell death (Globinska et al., 2014). Recent researches show that T lymphocytes could produce miRNAs transferred to DCs in the form of exosome miRNA, regulating gene expression of recipient cells in the process of antigen presentation. miRNA-containing exosome, passing on messages by circulating vesicles, is the third approach of intercellular communication with the exception of cell contact-depending and soluble molecules-mediated signaling, and it also takes part in antigen presentation, cell migration and differentiation to regulate immune responses (Okoye et al., 2014; Robbins and Morelli, 2014). Among which, miR-21, miR-150, miR-320, and Let-7 family are the more common exosomes.

About 2500 miRNAs have been detected in human peripheral blood, and a great deal of which have a cell-specific expression pattern. In physical conditions, there is a significant difference of miRNAs distribution in the blood, serum, plasma and exosome, even in different cell subsets. Among them, expression of miRNA precursors in neutrophils, monocytes and NK cells is quite rich, whereas CD4+ and CD8+ cells express the least (Juzenas et al., 2017). Phagocytes have a rich membrane system and could produce multiple miRNAs-containing exosomes. But there is still scarce knowledge about the way of these exosomes to participate in oriented differentiation of lymphocytes in the process of antigen presentation. Human miR-let-7 family plays an important role in the regulation of cell differentiation by directly or indirectly targeting genes related to cell cycle (Ambros, 2004; Roush and Slack, 2008). Let-7 family contains 13 isoforms, whose gene sequence and function are highly conserved in various animal species (Lee et al., 2016). Self-renewal could be inhibited in DGCR8 deletion embryotic stem cell when let-7 was inserted (Adlakha and Seth, 2017). In addition, let-7 inhibition could promote the transformation from un-differential somatic cells to induced pluripotent stem cells (iPSCs). Also, miRNA let-7b regulates neural stem cell proliferation and differentiation via interaction with stem cell regulator TLX and cell circle regulator CyclinD1 (Zhao et al., 2010). Since then, let-7 family is of great importance in self-differentiation and self-renewal of stem cells acting as regulators. Furthermore, miR-let-7d produced by Treg cells was released in the form of exosome and then transmitted to Th1 cells to suppress its proliferation and function (Okoye et al., 2014). Therefore, RSV infection induces a lot of differential expression miRNAs, which not only participate the acute stage inflammatory reaction, but act as triggers to the dysfunction of immunological bias, which might correlate to the recurrent wheezing post virus infection in later life.

## miRNAs Are Potential Components Associated With the Development of Recurrent Wheezing Evoked by RSV Infection

Combined with the above mentioned, it could be inferred that there are two vital pathological basis of wheezing recurrence after RSV infection: firstly, RSV acute infection promotes the initiation of inflammatory programs mediated by macrophages and DCs, increased activity of LT synthetase as well as upregulation of LTRs. Secondly, aberrant differentiation and dysfunction of lymphocytes resulting in the immune bias could contribute the recurrent wheezing. During the pathological process, differential expression of miRNAs and/or exosomes induced by RSV replication could be the initial inducers and bridge factors.

**FIGURE 4:**
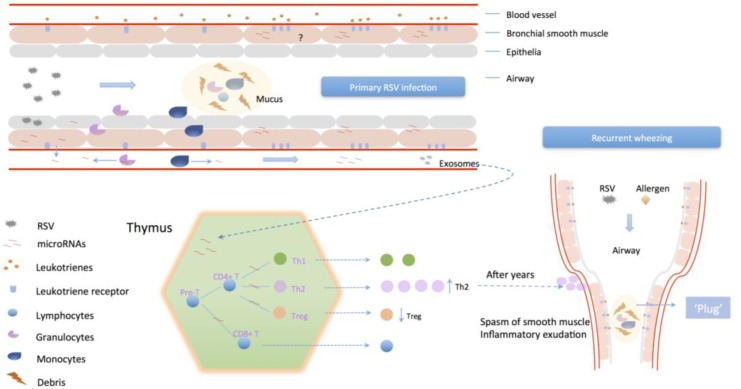
The potential mechanism of primary RSV infection associates to recurrent wheezing. RSV infection could enhance the intensity of reaction to inflammatory mediators in airway smooth muscle via differential miRNAs expression, which regulate the activity of LTs/PGs synthetase and upregulate their receptors distribution. Also, modulation of lymphocyte differentiation by miRNAs or exosomes could promote Th2 cells differentiation and proliferation and impair Treg cells to inhibit host immune tolerance, leading to enhanced sensitization of exogenous stimulus.

In the circumstance that viruses were cleared by host immune system after RSV infection, respiratory tissues and lymphocytes still maintain the state of hyper-reactivity and chronic inflammation (**Figure 4**) (Obando-Pacheco et al., 2018; Simoes et al., 2018). And the over-expression of inflammatory mediators and their receptors promote the response intensity to exogenous stimulus. Infant wheezing correlated to RSV infection is obviously age-specific. Children under age of 5 have an immaturity of lymphocyte distribution and function, the predominance of inflammation related to Th2 response, and the function decline of Treg cells, which could be corrected with age (Christiaansen et al., 2016). On account that RSV induces differential expression of miRNAs, and activity of LT syntheses is under regulation of specific miRNAs (Busch et al., 2015), it could be inferred that miRNAs induced by primary RSV infection contribute to the high-sensitivity to allergen. So far, there are still many obstacles to be clarified. For example, whether the continuous aberrant expression of miRNAs after RSV infection has an influence in unphysiologic alteration of inflammation factors expression in bronchial smooth cells, which leads to the state of inflammatory sensitization, or miRNAs are only the transient action on it, and when and how the immunologic bias is redressed with age. As for lymphocytes, particular for immature lymphocytes during early childhood, the aberrant expression of miRNAs might have an effect on lymphocyte differentiation and function. Acting as inducers, RSV infection could promote synthesis of inflammatory mediators and upregulation of LTRs in airway smooth muscle cells via regulation of miRNAs, and then increase the host reactivity to exogenous stimulus, which is related to wheezing severity. Modulation of lymphocyte differentiation by miRNAs or exosomes could reduce the host immune tolerance with enhanced sensitization of exogenous stimulus, which is related to wheezing frequency.

Differentiation of Treg cells could be suppressed by RSV infection, whereas the amount of CD4+CD25+Foxp3+ Treg cells reduces over age in premature infants. That the declining tendency could be improved by high-dose of intravenous immunoglobin (IVIG) (Liu et al., 2018), which is in accordance with the mechanism of immune regulation in clinical therapy that IVIG is used for the severe RSV infected pneumonia (Groothuis et al., 2011). RSV infection in early life has been considered as an independent high-risk factor of recurrent wheezing or asthma. However, for lack of knowledge of factors taking parts in the development of AHR, there has been less effective therapeutic strategies to prevent or retard the occurrence of later life wheezing (Fonseca et al., 2018).

## miRNAs Are Potential Targets of Designing miRNAs-Based Therapeutic Strategy for Viral Induced AHR

To date, miRNAs are potential therapeutic targets via either inhibiting viral genome or promoting host antiviral genes signaling (Peng et al., 2018), not only for RSV, but also for the other respiratory virus infections. New evidence has emerged on the potential role of miRNAs in influenza A virus (IAV)-induced host antivirus responses and in the modulation of the production of cytokines (Nguyen et al., 2018). For instance, miR-323-5p, miR-491-5p, and hsa-miR-654-5p directly bind to IAV H3N2 PB1 RNA, exerting an inhibitory effect on IAV replication in MDCK cells. Hsa-let-7c reduces viral replication by targeting and degrading M1 cRNA. hsa-miR-485-5p targets both RIG-I and viral PB1 to prevent spurious activation of antiviral signaling and suppress influenza virus infection. Treatment with the agomir delivery suppressed viral replication and effectively improved protection against lethal challenge with PR8 (H1N1) in mice. Besides, IAV infection decreases the expression of histone deacetylase 1 (HDAC1) that plays an important role in the activation of type-I IFN response against IAV infection. The use of miRNAs to target host factors that are utilized by viruses to promote infection and virus replication is a developing antiviral strategy, as it is hypothesized to overcome the selective pressure and subsequent drug resistance seen with direct virus-targeting antivirals (McCaskill et al., 2017). Host-targeting antiviral miRNAs could provide a complementary strategy for controlling infection, and they further illuminate host factors that are important in respiratory virus infections. Understanding the role of these small molecules in the antiviral immune response and identification of potential miRNAs-targeted genes may help to clarify the mechanisms of virus–host interaction and lead to the development of new antivirus treatments (Globinska et al., 2014). Targets of lead candidates, miR-124, miR-24, and miR-744, were identified within the p38 mitogen-activated protein kinase (MAPK) signaling pathway, and this work identified MAPK-activated protein kinase 2 as a broad-spectrum antiviral target required for both influenza and RSV infection. For three of these miRNAs, a portion of their antiviral activity was attributed to their suppression of the p38 MAPK pathway and MK2 in particular (Kim et al., 2017).

miRNAs play an essential role in the development of airway hyper reactivity, taking part in the regulation of inflammatory programs and induction of immune tolerance reduction. Nevertheless, identification of pathological basis needs further studies. In ALRI cases with RSV infection, detection of certain miRNAs expression may become a novel method to evaluate the severity of illness, which probably have the guiding significance to severity rating, especially in preterm newborn cases. Also, targeting specific miRNAs may be the potential prevention method or therapeutics strategy for infant wheezing related to RSV infection. Therefore, miRNAs target on the regulation of LTs synthesis and lymphocytes polarization will be promising treatment strategy for RSV-related AHR in future, and the strategy could also be applied to other respiratory virus infections. However, whether those miRNAs expression caused by RSV in acute stage infection act as transient or persistent impact on ARH is need further study in future.

## Conclusion

In summary, in this review, we discuss the miRNAs differential expression caused by RSV infection, which are involving in interfering LTs synthesis enzyme activity, deciding the immune cells polarization and causing immunological homeostasis bias, contribute to ARH in later life related to post-RSV infection. miRNAs could be the potential bio-marker of detecting severe infection and therapeutic targets for RSV.

## Author Contributions

SF collected references, drawn the figures, and wrote this paper. DZe and JZ participated in the writing and discussion. DZh wrote and organized this paper.

## Conflict of Interest Statement

The authors declare that the research was conducted in the absence of any commercial or financial relationships that could be construed as a potential conflict of interest.
